# The distribution of three candidate cold-resistant SNPs in six minorities in North China

**DOI:** 10.1186/s12864-018-4524-1

**Published:** 2018-02-12

**Authors:** Qiuyan Li, Kexian Dong, Lidan Xu, Xueyuan Jia, Jie Wu, Wenjing Sun, Xuelong Zhang, Songbin Fu

**Affiliations:** 1 Laboratory of Medical Genetics, Harbin Medical University, 157 Baojian Road, Nangang District, Harbin, 150081 China; 2 Key Laboratory of Medical Genetics, (Harbin Medical University), Heilongjiang Higher Education Institutions, 157 Baojian Road, Nangang District, Harbin, 150081 China; 3 Editorial Department of International Journal of Genetics, Harbin Medical University, Harbin, China

**Keywords:** Minorities, CPT1A, FADS, SNP, North China

## Abstract

**Background:**

Heilongjiang Province located in northeast China is a multi-ethnic region with people who have lived in cold conditions for several generations. Fatty acids are important to people with cold resistance. CPT1A encodes a protein that imports long-chain fatty acids into the mitochondria for fatty-acid oxidation. FADS is an essential enzyme for the synthesis of long-chain polyunsaturated fatty acids.

**Results:**

In the present study, we investigated the distributions of three cold resistance-related SNPs (rs80356779 G > A in CPT1A, rs7115739 T > G in FADS3 and rs174570 C > T in FADS2) from six populations that included 1093 individuals who have lived in Heilongjiang Province for at least three generations. The frequencies of rs174570 and rs7115739 were different in our six north minorities compared to the Chinese Dai in Xishuangbanna (CDX) in southern China. All the SNPs in Hezhen were significantly different from other five studied populations. In addition, the genetic distribution of rs174570 in Daur was significantly different from Manchu and Korea, and the frequency of rs7115739 in Ewenki was significantly different from the other populations. The results also showed that the frequencies of the three SNPs in the six minorities were different from those of Greenlandic Inuit and Siberian population.

**Conclusions:**

Our results showed the distributions of the three cold resistance-related SNPs from six populations that included 1093 individuals in northern China. Distributions of the allele frequencies for the cold resistance-related SNPs in northern China were statistically different from those in southern China. These data help to establish the DNA genome database for the six populations and fully preserve existing minority genetic information.

**Electronic supplementary material:**

The online version of this article (10.1186/s12864-018-4524-1) contains supplementary material, which is available to authorized users.

## Background

Many studies have suggested that there are different genetic structures in different populations due to their complex demographic histories [1]. China is a multi-ethnic country with a long history of complex national integration and migration. These ethnic origins and mutual relations can be explored from various aspects, such as archeology, language, culture, physical characteristics, etc. Under same environmental condition, different populations can evolve to form similar physical characteristics. Although the size of the gene affected by the environment is very small, it can be transmitted and stabilized from generation to generation to reflect important evolutionary events, forming the unique genetic diversity of the nation.

Having lived in northern China for a long period of time, Daur and other minorities have been subjected to cold climates. Thus, there may be some physiological differences contributing to their cold adaptations. Non-shivering thermogenesis is a physiological mechanism that generates body heat through mechanisms aside from mechanical shivering [2], and it may have been selected in humans who live in low-temperature regions under cold stress. Brown adipose tissue is an important site for non-shivering thermogenesis, which burns fat and uncouples mitochondrial oxidative metabolism from ATP production to produce heat [3]. It has been known that brown adipose tissue exists in mammalian infants. However, a recent study detected brown adipose tissue in distinct deposits in adult humans and interspersed in deposits of white adipose tissue. Since metabolic rates are known to be higher among humans living in extreme northern regions, metabolic rates in the non-shivering thermogenesis pathway in brown adipose tissue may have been subject to selection in response to cold stress [4].

Carnitine palmitoyltransferase 1 (CPT1) is a key regulatory enzyme that regulates fatty acid oxidation and has three isoforms, CPT1A, CPT1B and CPT1C. The CPT1A gene encodes a protein that imports long-chain fatty acids into the mitochondria for fatty acid oxidation. This mechanism is helpful for maintaining energy homeostasis when carbohydrate intake is insufficient [5]. CPT1B is expressed mainly in muscle. Different from CPT1A and CPT1B, CPT1C does not catalyze acyltransferase activity by using prototypic substrates [6]. Biochemical analyses using metabolomics-based screening strategies have clearly shown that mitochondrial activity is regulated by CPT1, and CPT1A is also a target for oxidative stress in human cells [7]. Ten missense variants were found in the carnitine acyltransferase gene family (CPT1A, CPT1B, CPT1C, CPT2, CRAT and CROT) that were related to fatty acid metabolism including CPT1A p.P479L (rs80356779). A study on Northeast Siberian populations showed that the CPT1A mutation conferred a metabolic advantage for Northeast Siberian populations for adaptation to their traditional high-fat diet [8].

The Fatty acid desaturase (FADS) genes, including FADS1, FADS2 and FADS3, code for enzymes that introduce double bonds at specific positions in a fatty acid chain [9]. In humans, the FADS genes are clustered within a 100-kb region on the long arm of human chromosome 11 (11q12–13.1) [10], which is a genomic cancer hot spot [11]. FADS1 (D5 desaturase) and FADS2 (D6/D8/D4-desaturase) have specificity for several fatty acid substrates [12–15]. FADS1 and FADS2 genes encode delta-5 desaturase (D5D) and delta-6 desaturase (D6D), respectively, which are essential enzymes for the synthesis of long-chain polyunsaturated fatty acids (LCPUFAs) [16]. FADS3 is a putative desaturase according to its sequence similarity with FADS1 and FADS2. The function of FADS3 is still unknown; however, its alternative transcripts can be directly upregulated by LCPUFAs via a PPARγ-dependent mechanism independent of the regulation of FADS1 or FADS2 [17].

Previous study identified a missense mutation (rs80356779; c.1436C > T: p.P479U) in CPT1A as the most likely causative variant for positive selection in Northeast Siberians, using whole-genome high-coverage sequence data [8]. FADS genes have been confirmed to be under selection in Inuit who live under challenging conditions in the Arctic [18] and have been hypothesized in other populations [19], suggesting that FADS genes are keys in human adaptation to cold. Intron variants in FADS2 (rs174570; NM_001281501.1: c.142-8038C > T) and FADS3 (rs7115739; NM_021727.4: c.1287-380A > C) have been significantly associated with cold adaptation.

In the present study, we aimed to explore the distribution of three SNPs in six ethnic minorities. We hoped that these genetic resources could provide information on the origins of the human race, evolution, migration and other aspects.

## Methods

### Study populations

Six populations from Hezhen, Daur, Manchu, Korean, Mongolian, and Ewenki, totaling 1093 individuals from Heilongjiang Province (around 45°44’N126°39′E), China were included in the study (Additional file 1: Figure S1). Among them, the Daur, Manchu and Ewenki participants were recruited from Qiqihar city, and the Hezhen, Korean, Mongolian participants were recruited from Jiamusi city located in Heilongjiang Province. All ethnic residents in the selected communities were screened through face-to-face survey for identifying individuals who were from a family without inter-ethnic marriage in the last three generations. We invited those who met the criteria to participate in the study. The study protocol was approved by the ethics committee of Harbin Medical University and all participants provided written informed consent for participating the scientific research of population genetic study.

### DNA extraction and genotyping

Genomic DNA was extracted from 200 μl of whole blood using the QIAamp blood kit (Qiagen, Hilden, Germany) according to the manufacturer’s protocol. The three cold resistance-related SNPs, rs80356779 G > A in CPT1A, rs7115739 T > G in FADS3 and rs174570 C > T in FADS2, were selected based on published articles [8, 18]. Genotyping was performed using the SNPscan™ Kit (Genesky Biotechnologies Inc., Shanghai, China) according to the manufacturer’s instructions and based on a high-throughput SNP genotyping method utilizing double ligation and multiplex fluorescence PCR.

### Database data

The genotype data of individuals from the three populations in China were downloaded from the 1000 genomes Project at https://www.ncbi.nlm.nih.gov/variation/tools/1000genomes/. The three populations included following individuals: (1) 93 Han Chinese in Beijing (CHB) individuals in northern China (around 39°55’N116°27′E); (2) 103 Chinese Dai in Xishuangbanna (CDX) individuals in southern China (around 22°3’N100°49′E); and (3) 105 Southern Han Chinese in China (CHS) individuals in southern China (around 30°22’N103°26′E).

### Statistical analysis

Hardy-Weinberg equilibrium of the controls was calculated using the *Chi-square* test. Fst calculations were performed using POPGENE (version 1.32) to assess the genetic differences among the six populations. Shannon’s information index was used to estimate the genetic diversity and the proportion of genetic variation within and between populations. The genotyping data have been submitted to Treebase under study number 22170 (https://treebase.org/treebase-web/user/summary.html?id=22170). All statistical analyses were performed using the SPSS 23.0 software (IBM-SPSS, Inc., Chicago, IL, USA). A probability (*P*) value < 0.05 was considered statistically significant.

## Results

### Genotyping data and Hardy-Weinberg test

The allele frequencies of three SNPs are shown in Table 1. A total of 1093 individuals, including 146 Hezhen, 228 Daur, 212 Manchu, 244 Korean, 155 Mongolian and 108 Ewenki individuals were investigated. The genotype frequencies in the study are summarized in Table 1. The *Chi-square test* suggested that the three polymorphisms followed Hardy-Weinberg equilibrium in the overall population (*P* > 0.05).

**Table 1:** The genotype distribution and Hardy-Weinberg equilibrium test for the three SNPs in six ethnic populations from China

Locus	AA	AB	BB	*Chi-square*	*P* value
rs174570	392	527	174	0.016	0.900
rs7115739	686	361	46	0.025	0.874
rs80356779	1063	30	0	0.204	0.651

*AA* wild homozygote, *AB* heterozygote, *BB* mutant homozygote

### The frequencies of the polymorphisms among different populations

As shown in Table 2, the heterozygosity statistics for all three SNPs were found to be highly similar among the Manchu, Korean and Mongolian individuals, showing that differences among the three populations were not significant. However, all the SNPs in the Hezhen population were significantly different from those in other five populations (*P* < 0.05). Daur were significantly different with Manchu and Korean regarding rs174570 genetic distribution (*P* < 0.05). The frequency of the rs7115739 polymorphism in Ewenki was significantly different from the other populations (*P* < 0.05).

**Table 2:** Summary of the heterozygosity statistics for all the loci in six ethnic populations from China

Ethnic	Locus	Sample size	Obs_Hom^d^	Obs_Het^d^	Exp_Hom^d^	Exp_Het^d^	Nei	Ave_Het^d^
Hezhen	rs174570	292	0.2877	0.7123	0.4983	0.5017	0.5000	0.4734
	rs7115739	292	0.4795	0.5205	0.5775	0.4225	0.4211	0.3370
	rs80356779	292	0.7945	0.2055	0.8150	0.1850	0.1884	0.0307
	Mean	292	0.5205	0.4795	0.6302	0.3698	0.3685	0.2804
	St. Dev		0.2559	0.2559	0.1648	0.1648	0.1643	0.2267
Daur	rs174570	456	0.5044^a^	0.4956	0.5049	0.4951	0.4940	0.4734
	rs7115739	456	0.6886^a,c^	0.3114	0.6960	0.3040	0.3033	0.3370
	rs80356779	456	1.0000^a^	0.0000	1.0000	0.0000	0.0000	0.0307
	Mean	456	0.7310	0.2690	0.7336	0.2664	0.2658	0.2804
	St. Dev		0.2505	0.2505	0.2497	0.2497	0.2491	0.2267
Manchu	rs174570	424	0.5849^a,b^	0.4151	0.5632	0.4368	0.4357	0.4734
	rs7115739	424	0.7217^a,c^	0.2783	0.6844	0.3156	0.3149	0.3370
	rs80356779	424	1.0000^a^	0.0000	1.0000	0.0000	0.0000	0.0307
	Mean	424	0.7689	0.2311	0.7492	0.2508	0.2502	0.2804
	St. Dev		0.2115	0.2115	0.2255	0.2255	0.2250	0.2267
Korean	rs174570	488	0.5902^a,b^	0.4098	0.5831	0.4169	0.4160	0.4734
	rs7115739	488	0.7377^a,c^	0.2623	0.7597	0.2403	0.2399	0.3370
	rs80356779	488	1.0000^a^	0.0000	1.0000	0.0000	0.0000	0.0307
	Mean	488	0.7760	0.2240	0.7809	0.2191	0.2186	0.2804
	St. Dev		0.2076	0.2076	0.2092	0.2092	0.2088	0.2267
Mongolian	rs174570	310	0.5419^a^	0.4581	0.5037	0.4963	0.4947	0.4734
	rs7115739	310	0.6903^a,c^	0.3097	0.6354	0.3646	0.3635	0.3370
	rs80356779	310	1.0000^a^	0.0000	1.0000	0.0000	0.0000	0.0307
	Mean	310	0.7441	0.2559	0.7130	0.2870	0.2860	0.2804
	St. Dev		0.2337	0.2337	0.2571	0.2571	02563	0.2267
Ewenki	rs174570	216	0.5278^a^	0.4722	0.4977	0.5023	0.5000	0.4734
	rs7115739	216	0.6019^a^	0.3981	0.6186	0.3814	0.3796	0.3370
	rs80356779	216	1.0000^a^	0.0000	1.0000	0.0000	0.0000	0.0307
	Mean	216	0.7099	0.2901	0.7055	0.2945	0.2932	0.2804
	St. Dev		0.2540	0.2540	0.2622	0.2622	0.2609	0.2667

All data were calculated by POPGENE software

^a^Compared with Hezhen, *P* < 0.05

^b^Compared with Daur, *P* < 0.05

^c^Compared with Ewenki, *P* < 0.05

^d^Obs_Hom: observed homozygosity; Obs_Het: observed heterozygosity; Exp_Hom: expected homozygosity; Exp_Het: expected heterozygosity; Ave_Het: Average heterozygosity

We also compared the allele frequency distributions in the current minorities and other populations, including one population located in northern China (CHB) and two located in southern China (CDX and CHS) (Additional file 2: Table S1). Tables 3 and (Additional file 2: Table S2) showed that the rs174570 and rs7115739 allele frequencies were different among the populations (*P* < 0.05). The frequencies of rs174570 ranged from 0.5000 to 0.7049 in six minorities. The frequencies in Korean and Manchu were similar, but different with other four minorities, and the frequencies of rs174570 were different in our six minorities from the CDX, which is the southeast population in this study. In addition, the frequencies of rs7115739 were different in the CDX when compared with most of our six minorities except for Hezhen, and there were strong differences between Hezhen and other populations in northern China, including Daur, Manchu, Korean and CHB (*P* < 0.05). The allele frequency in Korean was also different from that of other ethnic groups. However, in our six minorities, the A-allele in rs80356779 polymorphism was only detected in Hezhen, with the frequency of 0.1027.

**Table 3:** The *P* values for rs174570 compared between six minorities in northern China and three populations in southern China

Population	Hezhen	Daur	Manchu	Korean	Mongolian	Ewenki	CDX	CHB	CHS
Hezhen	–	2.150	23.271	32.845	1.607	0.011	34.346	10.398	0.011
Daur	0.143	–	26.918	38.388	1.707	0.003	38.657	11.341	3.438
Manchu	< 0.001	< 0.001	–	0.703	12.451	18.525	104.581	0.707	38.016
Korean	< 0.001	< 0.001	0.402	–	19.467	26.181	123.615	2.369	49.262
Mongolian	0.205	0.191	< 0.001	< 0.001	–	1.129	48.659	4.520	8.179
Ewenki	0.918	0.959	< 0.001	< 0.001	0.288	–	31.780	8.570	2.796
CDX	< 0.001	< 0.001	< 0.001	< 0.001	< 0.001	< 0.001	–	67.864	16.470
CHB	0.001	0.001	0.401	0.124	0.034	0.003	< 0.001	–	20.560
CHS	0.918	0.064	< 0.001	< 0.001	0.004	0.095	< 0.001	< 0.001	–

Note: *χ*2 value (above diagonal) and *P* value (below diagonal)

*CDX* Chinese Dai in Xishuangbanna, China, *CHB* Han Chinese in Beijing, China, *CHS* Southern Han Chinese

### Analysis of population genetic structures

**Fig. 1:**
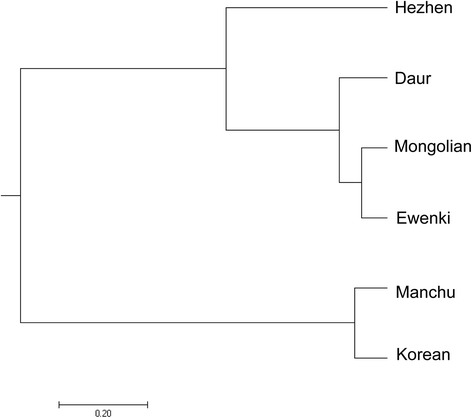
Dendrogram based on Nei’s genetic distance. The phylogenetic tree was created using a modified UPGMA method from the NEIGHBOR procedure in PHYLIP version 3.5

As shown in Table 4, the Fst values of the SNPs among the six populations varied from 0.0160 to 0.0871. According to analysis of gene diversity, the genetic differentiation Fst value among the populations was − 0.0244, which indicates that there was 97.56% genetic variation within the populations and that the genetic differentiation was low and found mainly within the population caused by individuals in each group. The average Nm was 9.9867, and large gene flow was the main reason for the low level of genetic differentiation. As shown in Table 5, the genetic distance among the populations ranged from 0.0011 to 0.0322; the genetic differentiation among the populations was not significant. The dendrogram produced with UPGMA based on Nei’s genetic distances assessed the relationships among the populations (Fig. 1). The six populations were divided into two main branches, one comprised of four populations, including Hezhen, Daur, Mongolian and Ewenki, and the other comprised of two populations, Manchu and Korean.

**Table 4:** Summary of the F-Statistics and gene flow for all the loci

Locus	Sample size	Fis	Fit	Fst	Nm^a^
rs174570	2186	−0.0432	−0.0154	0.0267	9.1188
rs7115739	2186	−0.0288	−0.0124	0.0160	15.4225
rs80356779	2186	−0.1145	− 0.0174	0.0871	2.6200
Mean	2186	−0.0397	−0.0143	0.0244	9.9867

^a^Nm: Gene flow estimated from Fst = 0.25 (1-Fst)/Fst

**Table 5:** Nei’s unbiased measures of genetic identity and genetic distance

Populations	Hezhen	Daur	Manchu	Korean	Mongolian	Ewenki
Hezhen	–	0.9897	0.9776	0.9683	0.9934	0.9952
Daur	0.0103	–	0.9931	0.9896	0.9988	0.9969
Manchu	0.0226	0.0070	–	0.9985	0.9920	0.9849
Korean	0.0322	0.0105	0.0015	–	0.9861	0.9773
Mongolian	0.0067	0.0012	0.0080	0.0140	–	0.9989
Ewenki	0.0048	0.0031	0.0152	0.0230	0.0011	–

Note: Nei’s genetic identity (above diagonal) and genetic distance (below diagonal)

## Discussion

Molecular studies have been effective methods in exploring the geographic origins of humans in the last few decades. Populations living in the same region may have different origins but should have similar phenotypes due to the similar environment. A study detected the allele frequencies of SNPs in metabolic genes among 52 worldwide populations using the Bayesian likelihood test. Strong signals were found for FADS2 and FADS3, which are involved in lipid metabolism, suggesting a relationship between genetic variants and cold resistance [20]. CPT1 is the key enzyme in carnitine-dependent transport across the mitochondrial inner membrane, and its deficiency results in a decreased rate of fatty acid β-oxidation.

The six populations examined in this study have lived in Heilongjiang Province situated in northern China for hundreds of years; the climate in this area is very cold with temperatures below − 25 °C in the winter. The people who live here may have experienced cold adaptations and genetic variations that generate physiological differences through metabolic pathways. CPT1A and FADS participate in the metabolism of fatty acids, which can store energy and heat for cold resistance. To live in a cold environment, people had to consume food containing much more fat in their daily diets. Furthermore, this has been reported in other cold-resistant populations. Therefore, CPT1A and FADS may be important factors for cold adaptation.

In this study, we investigated the diversity of cold resistance-related genes among six minorities from the northeast region of China to explore the hereditary differences among six populations. We selected three SNPs from adipose metabolism-related genes for the analysis. We also compared the allele frequencies between our study cohort and other populations in China. The *Chi-square* test suggested that the three polymorphisms followed Hardy-Weinberg equilibrium in the overall populations, although the SNPs in several minorities deviated from HWE (not shown). We found that the frequencies of our six minorities were similar to CHB, although there were some differences among them. However, when compared with populations located in southern China, great differences were observed. Since the CHB and six minorities are all located in northern China, and CHS and CDX are both located in southern China, the genetic differences between the CHB and six minorities are much smaller than those between the minorities and CHS and CDX. Thus, we presume that the gene may be undergoing strong selective pressures to resist cold and adapt to other environments. Additionally, there were strong differences between the six populations and the Greenlandic Inuit and Siberian population in allele frequencies. The allele frequencies of populations in northern China were in the middle of populations in southern China and Greenlandic Inuit and Siberian population, and there was a tendency to change with the latitude. In addition, Finnish in Finland (FIN) and British in England and Scotland (GBR) who live in northern area with an latitude around 55°N were examined to find the frequencies of rs174570 and rs7115739 polymorphisms in the 1000 Genomes Project. The frequencies of rs174570 in FIN and GBR are 0.2525 and 0.1868, respectively. The frequencies of rs7115739 in FIN and GBR are 0.9495 and 0.9505, respectively. These frequencies are very similar to those of Northeast Siberians and the Inuit, indicating that rs174570 and rs7115739 polymorphisms are associated with adaptation to cold. The results suggest that different minorities display unique genetic diversity in the nation during its long historical evolution for adapt to the cold environment.

The heterozygosity statistics for all three SNPs were found to be highly similar among Manchu, Korean, and Mongolian individuals, showing that the differences among the three populations were not significant. However, all the SNPs in the Hezhen population were significantly different from the other five populations. Daur was significantly different from Manchu and Korean with respect to the rs174570 genetic distribution. The frequency of the rs7115739 polymorphism in Ewenki was significantly different from the other populations. The differences may be associated with genetic drift.

To measure the genetic differentiation, the Fst values were calculated for each SNP to quantify the differences among the populations. The results showed that the genetic differentiation among the populations was not significant. To assess the relationships among populations, a dendrogram was produced by UPGMA. The dendrogram divided the six populations into two main branches: one comprised of four populations, including Hezhen, Daur, Mongolian and Ewenki, and the other comprised of two populations, Manchu and Korean. The occurrence of this cluster relationship is consistent with a previous published study [21]. According to ancient literature, the Hezhen absorbed multi-ethnic elements as it was a nation of multi-sources; some clans even absorbed the Mongols. Recent studies have shown the genetic distance between the Hezhen nationality and Mongolians. The origin of the Daur population is called the theory of the “Mongolia branch.” After establishment of P.R. China, the Ewenki divided from the Mongolians and formed a nation. However, the current cluster relationship still needs to be confirmed, which requires more genome data combined with archaeology data and population history for further analysis and verification.

## Conclusions

In the present study, we investigated the distributions of three cold resistance-related SNPs from six populations that included 1093 individuals in northern China. Our results showed, for the first time, that the six minorities in northern China exhibit genetic differences in certain cold resistance-related genes when compared with each other and with southern Chinese populations, especially with CDX. These results highlight that ethnicity is a critical factor in the distribution of allele frequencies. Meanwhile, our study also contributed to the DNA genome database for the six populations.

## Additional files

Additional file 1: Figure S1. The location of populations. The six minority populations from Hezhen, Daur, Manchu, Korea, Mongolian and Ewenki are located in Heilongjiang Province (around 45°44’N126°39′E). CHB indicates Han Chinese in Beijing (around39°55’N116°27′E). CHS indicates Southern Han Chinese located in Sichuan province (around 30°22’N103°26′E). CDX indicates Chinese Dai in Xishuangbanna (around 22°3’N100°49′E). Greenlandic Inuit population live in area around 71°43’N42°24’W. Northeast Siberian population live in the northeast of Russia (around 78°50’N112°50′E). (DOCX 109 kb)

Additional file 2: Table S1. The frequencies of three polymorphisms in 11 populations **Table S2** The *P* values for rs7115739 compared between six minorities in northern China and three populations in southern China. (DOCX 16 kb)

## Abbreviations

CDX: Dai in Xishuangbanna
CHB: Han Chinese in Beijing
CHS: Southern Han Chinese in China
CPT1: Carnitine palmitoyltransferase 1
FADS: Fatty acid desaturase
