# Supplementary material for: The distribution of three candidate cold-resistant SNPs in six minorities in North China
Source: BMC Genomics. 2018 Feb 12;19:134. doi: 10.1186/s12864-018-4524-1 (PMC5809914; doi:10.1186/s12864-018-4524-1)
Supplement: Supplementary file 1 — The location of populations. The six minority populations from Hezhen, Daur, Manchu, Korea, Mongolian and Ewenki are located in Heilongjiang Province (around 45°44’N126°39′E). CHB indicates Han Chinese in Beijing (around39°55’N116°27′E). CHS indicates Southern Han Chinese located in Sichuan province (around 30°22’N103°26′E). CDX indicates Chinese Dai in Xishuangbanna (around 22°3’N100°49′E). Greenlandic Inuit population live in area around 71°43’N42°24’W. Northeast Siberian population live in the northeast of Russia (around 78°50’N112°50′E). (DOCX 109 kb) [file 12864_2018_4524_MOESM1_ESM.docx]

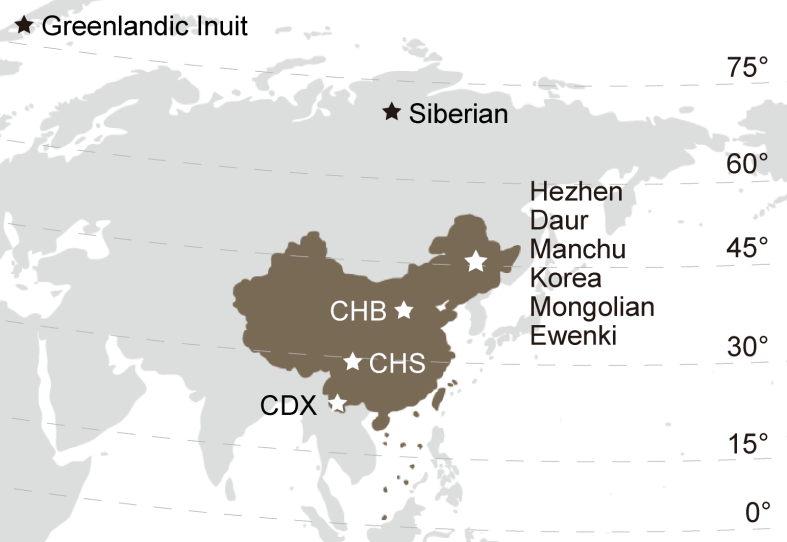


**Figure S1. The location of populations.**

The six minority populations from Hezhen, Daur, Manchu, Korea, Mongolian and Ewenki are located in Heilongjiang Province (around 45°44'N126°39'E). CHB indicates Han Chinese in Beijing (around39°55'N116°27'E). CHS indicates Southern Han Chinese located in Sichuan province (around 30°22'N103°26'E). CDX indicates Chinese Dai in Xishuangbanna (around 22°3'N100°49'E). Greenlandic Inuit population live in area around 71°43'N42°24'W. Northeast Siberian population live in the northeast of Russia (around 78°50'N112°50'E).
