# Supplementary material for: The distribution of three candidate cold-resistant SNPs in six minorities in North China
Source: BMC Genomics. 2018 Feb 12;19:134. doi: 10.1186/s12864-018-4524-1 (PMC5809914; doi:10.1186/s12864-018-4524-1)
Supplement: Supplementary file 2 — The frequencies of three polymorphisms in 11 populations Table S2 The P values for rs7115739 compared between six minorities in northern China and three populations in southern China. (DOCX 16 kb) [file 12864_2018_4524_MOESM2_ESM.docx]

**TableS1 The frequencies of three polymorphisms in 11 populations**

| **Populations** | **2n** | **rs174570** | |  | **rs7115739** | |  | **rs80356779** | |
| --- | --- | --- | --- | --- | --- | --- | --- | --- | --- |
|  |  | **C** | **T** |  | **G** | **T** |  | **G** | **A** |
| Hezhen | 292 | 0.5000 | 0.5000 |  | 0.6986 | 0.3014 |  | 0.8973 | 0.1027 |
| Daur | 456 | 0.5548 | 0.4452 |  | 0.8136 | 0.1864 |  | 1.0000 | 0 |
| Manchu | 424 | 0.6792 | 0.3208 |  | 0.8042 | 0.1958 |  | 1.0000 | 0 |
| Korean | 488 | 0.7049 | 0.2951 |  | 0.8607 | 0.1393 |  | 1.0000 | 0 |
| Mongolian | 310 | 0.5516 | 0.4484 |  | 0.7613 | 0.2387 |  | 1.0000 | 0 |
| Ewenki | 216 | 0.5046 | 0.4954 |  | 0.7454 | 0.2546 |  | 1.0000 | 0 |
| CDX* | 186 | 0.2312 | 0.7688 |  | 0.6667 | 0.3333 |  | - | - |
| CHB* | 206 | 0.6456 | 0.3544 |  | 0.7816 | 0.2184 |  | - | - |
| CHS* | 210 | 0.4238 | 0.5762 |  | 0.7190 | 0.2810 |  | - | - |
| Greenlandic Inuit†^[9]^ |  | 0.99 | 0.01 |  | 0.98 | 0.02 |  | - | - |
| Siberian†^[8]^ |  | - | - |  | - | - |  | 0.68 | 0.32 |

*Data downloaded from the 1000 genomes Project.

† Data from two publications.

**Table S2 The *P* values for rs7115739 compared between six minorities in northern China and three populations in southern China**

| **Population** | **Hezhen** | **Daur** | **Manchu** | **Korean** | **Mongolian** | **Ewenki** | **CDX** | **CHB** | **CHS** |
| --- | --- | --- | --- | --- | --- | --- | --- | --- | --- |
| Hezhen | - | 13.234 | 10.610 | 29.975 | 3.002 | 1.341 | 0.539 | 4.243 | 0.246 |
| Daur | <0.001 | - | 0.124 | 3.844 | 3.070 | 4.137 | 16.155 | 0.923 | 7.585 |
| Manchu | <0.001 | 0.724 | - | 5.226 | 1.965 | 2.933 | 13.505 | 0.441 | 5.865 |
| Korean | <0.001 | 0.050 | 0.022 | - | 12.796 | 13.801 | 32.554 | 6.649 | 19.780 |
| Mongolian | 0.083 | 0.080 | 0.161 | <0.001 | - | 0.174 | 5.230 | 0.286 | 1.174 |
| Ewenki | 0.247 | 0.042 | 0.087 | <0.001 | 0.676 | - | 3.000 | 0.763 | 0.376 |
| CDX | 0.463 | <0.001 | <0.001 | <0.001 | 0.022 | 0.083 | - | 6.501 | 1.275 |
| CHB | 0.039 | 0.337 | 0.507 | 0.010 | 0.593 | 0.382 | 0.011 | - | 2.167 |
| CHS | 0.620 | 0.006 | 0.015 | <0.001 | 0.279 | 0.540 | 0.259 | 0.141 | - |

Note: *χ*^2^ value (above diagonal) and *P* value (below diagonal)

CDX: Chinese Dai in Xishuangbanna, China

CHB: Han Chinese in Beijing, China

CHS: Southern Han Chinese
